# Development and Validation of a Rapid RP-HPLC-DAD Analysis Method for the Simultaneous Quantitation of Paclitaxel and Lapatinib in a Polymeric Micelle Formulation

**DOI:** 10.3797/scipharm.1507-03

**Published:** 2015-12-29

**Authors:** Ebrahim Saadat, Fatemeh Ravar, Pouya Dehghankelishadi, Farid A. Dorkoosh

**Affiliations:** 1 Department of Pharmaceutics, Faculty of Pharmacy, Tehran University of Medical Sciences, Tehran P.O. Box 14155-645, Iran; 2 Medical Biomaterials Research Center, Tehran University of Medical Sciences, Tehran 14399-56131, Iran

**Keywords:** Paclitaxel, Lapatinib, Validation, Pharmaceutics, HPLC

## Abstract

A robust and rapid analysis method was developed and validated for the simultaneous assay of paclitaxel (PTX) and lapatinib (LPT) in a polymeric micelle formulation as a novel drug delivery system using high-performance liquid chromatography (HPLC). The assay was performed using the C18 MZ-Analytical Column (5 μm, 150 × 4.6 mm, OSD-3) which was protected with the C18 pre-column (5 μm, 4.0 × 4.6 mm, OSD-3). The mobile phase was composed of acetonitrile and water (70/30; V/V) with a flow rate of 0.5 mL/min and detection wavelength of 227 nm. Accuracy was reported as the relative error and was found to be less than 6.8%. The interday assay was evaluated to be 3.22% and 5.76% RSD for PTX and LPT, respectively. The intraday precision was found to be at its maximum value of 5.83% RSD. The limit of detection for both PTX and LPT was found to be 1 µg/mL by means of the newly developed method. The limit of quantitation for PTX and LPT was found to be 5 µg/mL. The calibration curves for both drugs were linear in the concentration range of 5 to 80 μg/mL. *In vitro* release for both drugs from the polymeric micelle was evaluated using the newly developed analysis method.

## Introduction

**Fig. 1:**
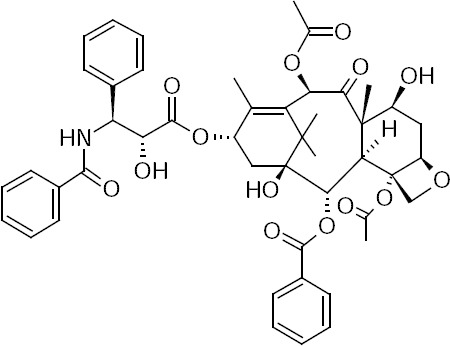
Chemical structure of PTX.

Paclitaxel (PTX) (Fig. 1) is a potent antitumor drug which was originally derived from the bark of the Pacific yew tree (*Taxus brevifolia*; family Taxaceae). It is indicated for various types of cancers including node-positive breast cancer, non-small cell lung cancer, advanced ovarian carcinoma, and Kaposi’s sarcoma [1, 2]. PTX inhibits cell proliferation by disturbing microtubule networks during mitosis [3]. PTX shows good clinical outcomes short–term, but the resistance of cancerous cells creates challenges in the treatment of tumors. Cancerous cells develop special pumps called P-glycoprotein, in which they pump out the drug molecules from the cell. This mechanism is known as efflux-mediated drug resistance which restricts the therapeutic efficacy of cytotoxic drugs [4]. Till now, several researches have been performed to inhibit these pumps by inhibitors and enhance the efficacy of the cytotoxic agents. Unfortunately, most of these pump inhibitors cause severe drug interactions and side effects which practically prevent them from being administered [5]. Recent studies suggest that tyrosine kinase inhibitors block efflux pumps by binding to the internal domain of such transporters.

**Fig. 2:**
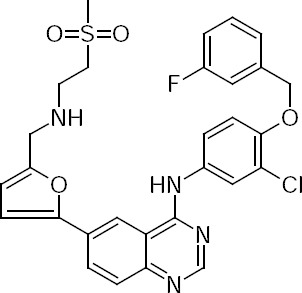
Chemical structure of LPT.

Lapatinib (LPT) (GW572016, Tykerb®) (Fig. 2) is an orally available selective dual tyrosine kinase inhibitor which inhibits both epidermal growth factor (EGFR) and Her-2 receptors that are overexpressed by cancer cells [6]. LPT is designed chemically as *N*-{3-Chloro-4-[(3-fluorobenzyl)oxy]phenyl}-6-[5-({[2-(methylsulfonyl)ethyl]amino}methyl)-2-furyl]quinazolin-4-amine. LPT is recommended in the treatment guidelines of Her-2 positive breast tumors and also in combination with PTX in metastatic breast cancer [7]. In the inhibition of efflux pumps, LPT reduces pumping out of the PTX molecules from cancerous cells and improves the cytotoxic efficacy. Recently, different drug delivery systems such as polyelectrolyte nano-capsules and lipopolymer micelles have been designed for co-delivery of PTX and LPT for ovarian and prostate cancers [8, 9].

Various analysis methods have been reported for the quantitative determination of PTX and LPT till now, but none of them analyzed both drugs simultaneously. For example, liquid chromatography electrospray tandem mass spectrometry [10] was developed for the quantitative determination of LPT in the biological samples. These methods are not applicable for the determination of LPT in pharmaceutical dosage forms and need serious modifications. Recently, an HPLC method was reported for the determination of LPT in pharmaceutical dosage forms, but it requires further optimization in terms of precision and accuracy which are the two main parts of method validation [11].

Moreover, several analysis methods have been developed and validated for the quantification of PTX [12–14]. However, as mentioned before, simultaneous analysis of PTX and LPT have not been reported till now. This method development is necessary for the simultaneous assay of both agents in various drug delivery systems such as polymeric micelles.

The aim of this study was to develop and validate a UV-HPLC method for the quantitative analysis of PTX and LPT simultaneously. This newly developed method was also applied to evaluate the release profile of PTX and LPT from the novel pluronic micelle.

## Experimental

### Chemicals and Reagents

LPT ditosylate (99.9% purity) was purchased from Beijing Mesochem Technology Co. (China) and used without further purification. PTX (99% purity) was obtained from Sinopharm Chemical Reagent Co. Ltd. (China). Pluronic F127 was purchased from Gilson Co. (France). Acetonitrile and methanol were purchased from Merck (Germany). All other reagents and solutions were from either HPLC or analytical grade and used as received. HPLC grade water was obtained from distilled water which passed through a water deionizer system and used freshly for the preparation of all solutions.

### HPLC Instrumentation and Conditions

Analyses of samples were performed by an HPLC system (Agilent Technologies^®^ Model 1260 Infinity) which was equipped with an autosampler (Agilent Technologies^®^, USA) fitted with a 25 μL sample loop, and G1315D diode array detector (Agilent Technologies^®^). Chromatographic data were monitored and analyzed by Chemostation for LC Systems software (B.04.02) (Agilent Technologies^®^). Samples were analyzed using the C18 MZ-Analytical Column (5 μm, 150 × 4.6 mm, OSD-3) protected with the C18 pre-column (5 μm, 4.0 × 4.6 mm, OSD-3). The mobile phase was composed of acetonitrile/water (70/30; V/V) with the flow rate of 0.5 mL/min. Samples were monitored at the detection wavelength of 227 nm. The column temperature was set at 25°C and 25 μL samples were injected onto the HPLC system every 30 min. The mobile phase was filtered prior to use by a PTFE filter (0.45 μm).

### Preparation of PTX/LPT Micelle Formulation

The PTX/LPT micelle was prepared by the thin film hydration method [15]. Briefly, 2.5 mg of PTX and 3.5 mg of LPT were dissolved in 10 mg/mL of pluronic solution in acetonitrile. The solution was poured into the round-bottom flask and evaporated at 60°C by a rotary evaporator (Buchi, Germany). The thin film was kept overnight under vacuum conditions and hydrated with 10 mL of water on the following day. In order to remove the unincorporated drug aggregates, the micelle dispersion was filtered through a modified cellulose acetate (CA) filter (0.45 µm). The filtered solution was lyophilized and kept at refrigerator temperature (2–8°C) for further experiments.

### Extraction of PTX/LPT from the Micelle Formulation

The lyophilized powder of the PTX/LPT micelle was reconstituted with 5 mL deionized water and 1 mL aliquot of the sample was withdrawn and diluted with 9 mL acetonitrile. The solution was sonicated with a probe-type ultrasonic sonicator (Hielscher, UP400S) with a power output of 180 watts and 5 second time intervals. After micelle disassembly, 1 mL of solution was centrifuged at 10000 rpm for 10 min using the Eppendorf micro-centrifuge (Brinkmann Inst. Inc., NY, USA). Twenty-five μl of the aliquot sample was injected directly onto the HPLC system.

### Preparation of Stock Solution

A stock solution containing a mixture of LPT and PTX with the concentration of 1 mg/mL was prepared in acetonitrile, respectively. The stock solution was wrapped up with aluminum foil to protect it from light and stored at 4°C. Aliquots of the standard stock solution of PTX and LPT were transferred into the 5-mL A-grade volumetric flasks by means of A-grade bulb pipettes. All solutions were made up to the volume with acetonitrile to final concentrations of 5, 10, 20, 40, and 80 μg/mL.

### Validation Procedure

The novel, developed HPLC method was validated according to the International Conference on Harmonization of Technical Requirements for Registration of Pharmaceuticals for Human Use [16]. This method was validated regarding linearity, limit of detection (LOD), limit of quantification (LOQ), accuracy, precision, specificity, and robustness.

#### Linearity

Linearity was analyzed at five different concentrations ranging from 5 µg/mL to 80 µg/mL. Each concentration was injected three times in order to obtain the area under the curve (AUC) which corresponded to each concentration. Accordingly, the AUC data were plotted versus PTX and LPT concentrations (µg/mL), separately. Linear regression analysis was assessed to determine the calibration equations [17]. Calibration equations were expressed as y= ax+b, for which a and b coefficients represent the slope and intercept of the curves, respectively.

#### Accuracy

Five replicates of 5, 40, and 80 µg/mL concentrations were analyzed to determine the closeness of the obtained results with the actual amounts. Accuracy was reported as the percent relative error (RE%) for each concentration.

#### Precision

Intraday and interday precisions are regarded as the major parameters for a validated analytical method. Intraday precision was conducted by analyses of samples with concentrations of 5, 40, and 80 µg/mL in replicates of five (every 2 hours). Interday precision was assessed by analyzing various concentrations (5, 40, and 80 µg/mL) in five replicates for three consecutive days. Both intraday and interday precisions were reported as the mean measured concentration along with the relative standard deviation (RSD).

#### Limit of Detection (LOD) and Limit of Quantification (LOQ)

The PTX/LPT binary standard mixture solution was diluted to determine the LOD and LOQ. The limit of detection (LOD) is defined as the minimum concentration which possesses a signal-to-noise ratio of three. The limit of quantification (LOQ) is defined as the minimum concentration that possesses a signal-to-noise ratio of ten [17].

#### Robustness and Ruggedness

As recommended by ICH guidelines, a robustness assessment was performed for the development of the validated analytical method [17]. Robustness indicates the ability of a method to tolerate small deliberate changes in the flow rate and mobile phase composition. Briefly, the flow rate was set at 0.4, 0.5, and 0.6 mL/min and the recoveries of drugs were analyzed as a response. The ratio of acetonitrile to water in the mobile phase composition (68/32, 70/30, and 72/28) was another parameter which was analyzed in the robustness study. Furthermore, some minor modifications in temperature and detection wavelength were applied. A ruggedness study was also carried out which confirms that the method is reproducible in different laboratories by different analysts.

### Stability Studies

The stability of samples was analyzed at two different temperatures. PTX/LPT binary mixtures (QC samples) were kept at refrigerator (2–8°C) and room temperature. After 24 hours, the samples were injected onto the HPLC system for further analyses. The concentrations of all samples were 40 µg/mL. The results of the stability studies were reported as the recovery percentage for PTX and LPT.

### The In Vitro Release Study of PTX and LPT from the Nano-Micellar System

An *in vitro* release study of PTX and LPT was performed by means of a dialysis method. One mL of the micellar solution which contained defined amounts of PTX and LPT was added to the dialysis bag (molecular weight cutoff of 12000 Da). The dialysis bag was completely end-sealed and fully immersed in 100 mL PBS (pH= 7.4) which contained 10% PEG 400 to enhance the solubility and maintain the sink condition. The *in vitro* release test was conducted at 37 ± 0.5°C on a shaker bath (50 rpm). At predetermined time intervals, 1 mL aliquots of the samples were withdrawn and filtered through a cellulose acetate syringe filter (0.22 μm) and analyzed by the above-mentioned method. After each sample’s removal, the entire release medium was substituted with fresh medium in order to maintain the sink condition.

## Results and Discussion

### HPLC Method Development and Optimization

For method validation and simultaneous analysis of PTX/LPT, various conditions such as different columns (C8 and C18) and mobile phase mixtures were tried. The C18 MZ-Analytical Column (5 μm, 150 × 4.6 mm, OSD-3) protected with the C18 pre-column (5 μm, 4.0 × 4.6 mm, OSD-3) at room temperature was found to be appropriate for the separation of both drugs efficiently. Different mobile phase mixtures including ammonium acetate with water and organic solvents including methanol and acetonitrile were tested. On the basis of preliminary experiments, a mobile phase composed of acetonitrile and water was selected for further analysis that showed good peak shape and resolution. Further attempts for mobile phase mixture composition showed that the mobile phase with a composition of 70% acetonitrile and 30% water (V/V) with the flow rate of 0.5 mL/min exhibited the appropriate separation of peaks. The optimum detection wavelength for the two compounds was obtained using the Agilent G1315D PDA detector. In this method validation, the isocratic mode was selected due to its simplicity and less involved variables which may potentially have affected the optimization process. The gradient method is not only time–consuming, but also makes variations between different columns and laboratories [18]. It has been reported that employing the gradient method led to some disturbances like baseline noise and also eluent mixing which can potentially influence the accuracy and precision of the method [19, 20]. The mentioned problems in the gradient method could be solved by the selection of a proper gradient elution system [21]. However, in sum, the isocratic method seems to be simpler and more user-friendly compared to the gradient method. Regarding the described condition above, all peaks of PTX and LPT were shaped well and free from tailing. The retention times were 9 and 17 min for PTX and LPT, respectively. According to the number of the column’s theoretical plates, there should be a minimum distance between the peaks to ensure that there is no overlap. The retention times demonstrated that both PTX and LPT peaks were separated efficiently by means of this newly developed method. Also, according to the literature, neither PTX-degraded products nor LPT-related derivatives have overlapped with the main peaks in the chromatogram [22, 23]. To identify the peak purity, diode array detection was also performed using peak purity software, which compared the spectra at the peak upslope, apex, and downslope. These results showed that the PTX and LPT peaks were homogenous and pure in all experiments. The chromatographic response of the stock solution in comparison to the freshly prepared solution showed no significant changes (<1%).

### Validation of the Method

#### Linearity

Linear calibration curves (n = 3) were obtained by plotting the peak areas (AUC) of PTX and LPT versus the concentration at five levels (5, 10, 20, 40, and 80 μg/mL) separately, each in triplicate. Linearity was determined by least-squares linear regression analysis of the obtained calibration curves [24]. Three correlation coefficients of R1 = 0.9968, R2 = 0.9960, and R3 = 0.9970 were obtained with the relative standard deviation (RSD %) values between 2.60 and 6.00 for PTX and correlations of R1= 0.9915, R2= 0.9920, and R3= 0.9923 with the relative standard deviation (RSD %) values between 0.06 and 3.80 for LPT. The equations for the calibration curve were typically calculated to be Y= 29.61X-29.50 for PTX and Y=32.99X+0.83 for LPT, in which Y is the area under the curve (AUC) and X corresponds to the concentration of each drug.

#### LOD and LOQ

The LOQ is defined as a signal-to–noise of more than ten-fold following three consecutive injections. The LOQ amounts for PTX and LPT in this validation method were found to be 5 µg/mL for both of the drugs. In these studies, the RSD% values for PTX and LPT were also calculated to be 2.60 and 3.17, respectively. The LOD amount is defined as a signal-to–noise of more than three. Regarding this definition, the LOD of PTX and LPT were found to be 1 µg/mL, in mixed solution.

#### Precision

Precision is an important factor in new method development and validation which indicates the repeatability of the obtained data on the same day (intraday precision) or other days (interday precision). Intraday precision was evaluated by injecting five independent PTX/LPT solutions at three different concentrations. Interday precision was carried out by injecting the same three samples over three consecutive days. Table 1 shows inter- and intraday precision of the developed method. The maximum RSD% values for interday precision for PTX and LPT were found to be 3.22% and 5.76%, respectively. The maximum RSD% for intraday precision was 3.70% and 5.83% for PTX and LPT, respectively. In the previous study which was validated for PTX by means of the HPLC method, the RSD% value was reported to be around 2.2% which did not differ significantly with this report. Although in the present work, two drugs were validated simultaneously [25]. Also, in other studies which were performed for validation of LPT by LC-MC/MC [26, 27] and UPLC-MC/MC [28], the percentages varied from 3.9 to 8.1 which were higher than that of the present method. Furthermore, previous studies which quantify LPT in the bulk pharmaceutical and tablet dosage form have reported precision RSD% of 0.509 and 0.223%, respectively. However, none of these studies were for simultaneous detection [11–29]. By applying the newly developed method, not only was a more precise analysis obtained, but also the simultaneous assay of two chemotherapeutic agents was provided.

**Tab. 1:**
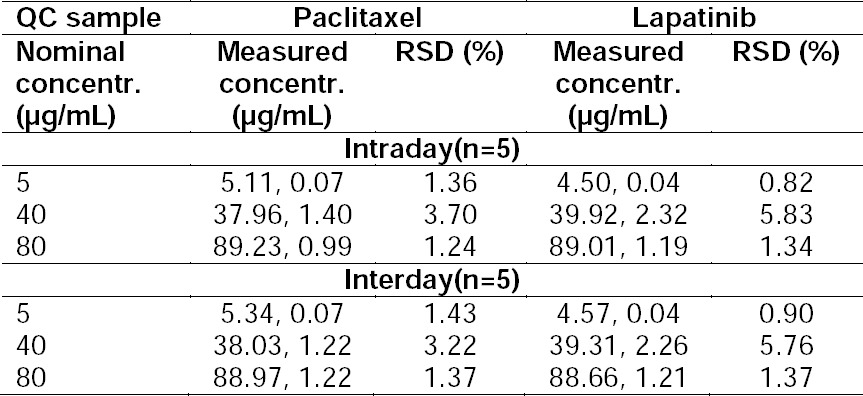
The Results of Inter- and Intraday Precision of QC Samples (n = 5, Mean, SD).

#### Accuracy

Accuracy is defined as the closeness of obtained data from an analysis method to the actual value or reference value. The accuracy of the PTX/LPT concentration of three levels at 5, 40, and 80 mg/mL from the calibration curve were determined in five replicates. Table 2 illustrates the accuracy data of PTX/LPT. In each series of data, the RE% was calculated and found to be less than 6.8% and 2.5% for PTX and LPT, respectively. In a previous method which was developed for the validation of PTX in pharmaceutical dosage form, the accuracy was explored. In that study, the RE% was reported to be around 2.5%. However, in the present study, PTX was validated simultaneously with LPT [23]. Also in another method, the accuracy of LPT alone was expressed as “recovery” which was reported to be in the range limit of 100 ± 4.1%. In that study, neither RE% nor the % RSD was reported [11].

**Tab. 2:**
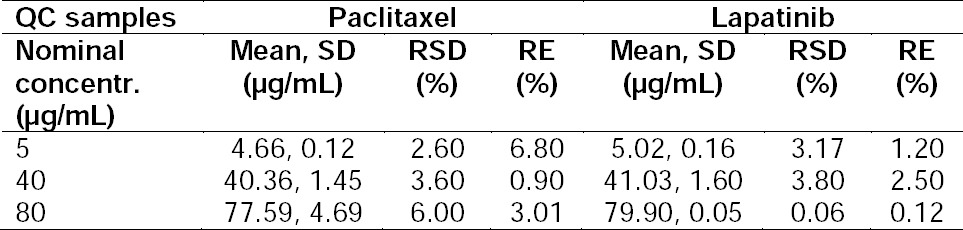
Accuracy Data of PTX/LPT Samples (n = 5, Mean, SD).

#### Ruggedness and Robustness Tests

Regarding the ICH guidelines, robustness is a substantial issue in the development of new analytical method validation [30]. Robustness of an analytical method is determined by applying changes in analysis conditions such as flow rate, the mobile phase composition, temperature, and detection wavelength [17]. The repeatability of the obtained data from these studies showed the robustness of the developed analytical method. Table 3 shows the recovery data of PTX/LPT under deliberate changes in the main factors of analysis. As seen in these series of data, there are no significant changes under the modified conditions (p-value < 0.05). Comparing the results from two different laboratories and two analysts, we confirmed the ruggedness of the new method. These results demonstrated that the method is not influenced by the laboratory and the analyst.

**Tab. 3:**
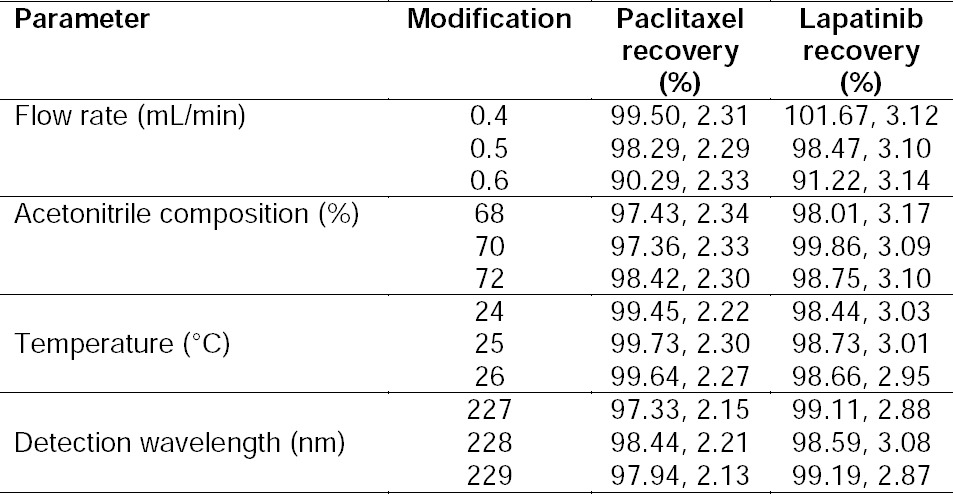
The Influence of Changes in the Experimental Conditions on the Response of the HPLC Method (n=3, Mean, SD).

### Stability Studies

**Fig. 3:**
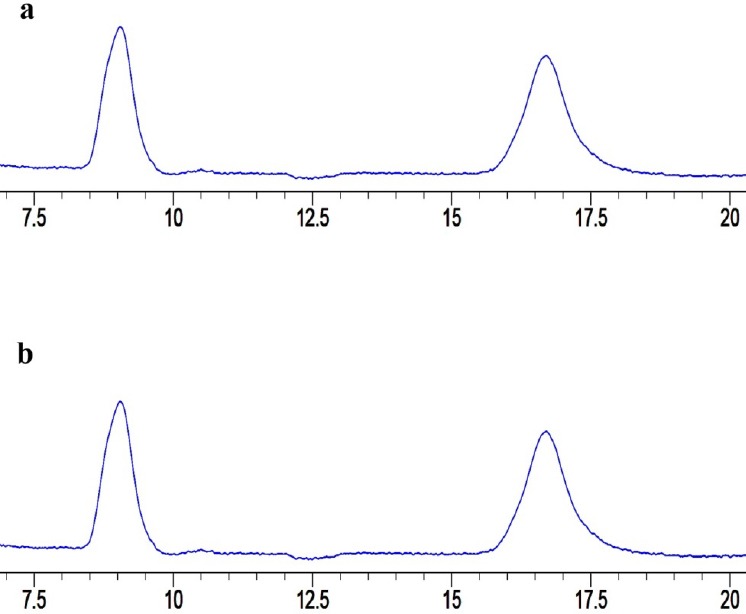
Chromatograms of PTX and LPT in a (refrigerated samples) and b (room temperature samples).

Stability studies were performed for three QC concentrations. Samples were incubated for 24 hours in the room (25°C) and refrigerator temperatures (2–8°C) and analyzed by means of the recovery percentage afterwards. According to the results which are summarized in Table 4, for both samples (room and refrigerator temperatures), no significant changes in recovery were found. Also, no significant changes in the resolution and retention times were detected for both drugs (Fig. 3).

**Tab. 4:**
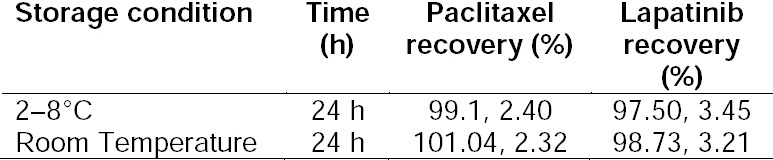
The Stability Study of Paclitaxel and Lapatinib in Different Temperatures (n=3, Mean, SD).

### In Vitro Release

**Fig. 4:**
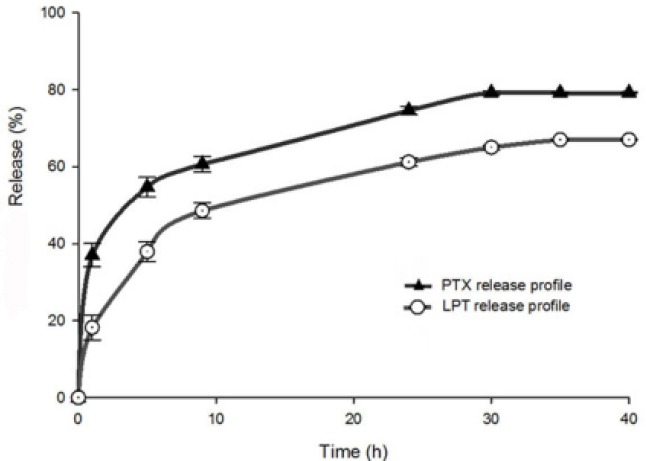
*In vitro* release profile of PTX and LPT in PBS (pH= 7.4, 37 ± 0.5°C, 50 rpm).

*The in vitro* release profile of the PTX/LPT-loaded micelle is shown in Fig. 4. In the first five hours of the release study, both chemotherapeutic agents demonstrated an initial burst release. After the initial burst release, a sustained release pattern was observed in the next 25 hours. LPT released more slowly in comparison to PTX due to its more hydrophobic characteristics. After 30 hours, both PTX and LPT reached the plateau phase. According to the obtained results from the release study, it can be concluded that the newly developed method is suitable for the determination of the PTX/LPT concentration in release media.

## Conclusion

A rapid, precise, and simple method was developed for simultaneous analysis of PTX and LPT by means of an HPLC method. The newly developed method showed acceptable precision and accuracy at least in the concentration range of 5 to 80 μg/mL. The developed method represented good resolution for both PTX and LPT. The validated analytical method is simple and reproducible which can be used in quality control departments. Moreover, by this method, the release profile of PTX and LPT from nano-micellar system was analyzed precisely.
